# An Update of Microsomal Prostaglandin E Synthase-1 and PGE_2_ Receptors in Cardiovascular Health and Diseases

**DOI:** 10.1155/2016/5249086

**Published:** 2016-08-09

**Authors:** Guangrui Yang, Lihong Chen

**Affiliations:** ^1^Institute for Translational Medicine and Therapeutics, University of Pennsylvania, Philadelphia, PA 19104-5158, USA; ^2^Advanced Institute for Medical Sciences, Dalian Medical University, Dalian 116044, China

## Abstract

Nonsteroidal anti-inflammatory drugs (NSAIDs), especially cyclooxygenase-2 (COX-2) selective inhibitors, are among the most widely used drugs to treat pain and inflammation. However, clinical trials have revealed that these inhibitors predisposed patients to a significantly increased cardiovascular risk, consisting of thrombosis, hypertension, myocardial infarction, heart failure, and sudden cardiac death. Thus, microsomal prostaglandin E (PGE) synthase-1 (mPGES-1), the key terminal enzyme involved in the synthesis of inflammatory prostaglandin E_2_ (PGE_2_), and the four PGE_2_ receptors (EP1–4) have gained much attention as alternative targets for the development of novel analgesics. The cardiovascular consequences of targeting mPGES-1 and the PGE_2_ receptors are substantially studied. Inhibition of mPGES-1 has displayed a relatively innocuous or preferable cardiovascular profile. The modulation of the four EP receptors in cardiovascular system is diversely reported as well. In this review, we highlight the most recent advances from our and other studies on the regulation of PGE_2_, particularly mPGES-1 and the four PGE_2_ receptors, in cardiovascular function, with a particular emphasis on blood pressure regulation, atherosclerosis, thrombosis, and myocardial infarction. This might lead to new avenues to improve cardiovascular disease management strategies and to seek optimized anti-inflammatory therapeutic options.

## 1. Introduction

Prostaglandin (PG) E_2_ is an important lipid mediator that regulates diverse and important physiological processes, such as gastric epithelial cytoprotection, renal blood flow maintenance, cardiovascular tone and blood pressure regulation, reproduction and parturition, bone formation, sleep, and neuroprotection. One of the major pathophysiological functions of PGE_2_ is to elicit actions such as pyrexia, pain sensation, and inflammation. Thus, the analgesic and anesthetic effects of the most widely used nonsteroidal anti-inflammatory drugs (NSAIDs) are thought to be driven by inhibition of the production of PGE_2_.

**Figure 1:**
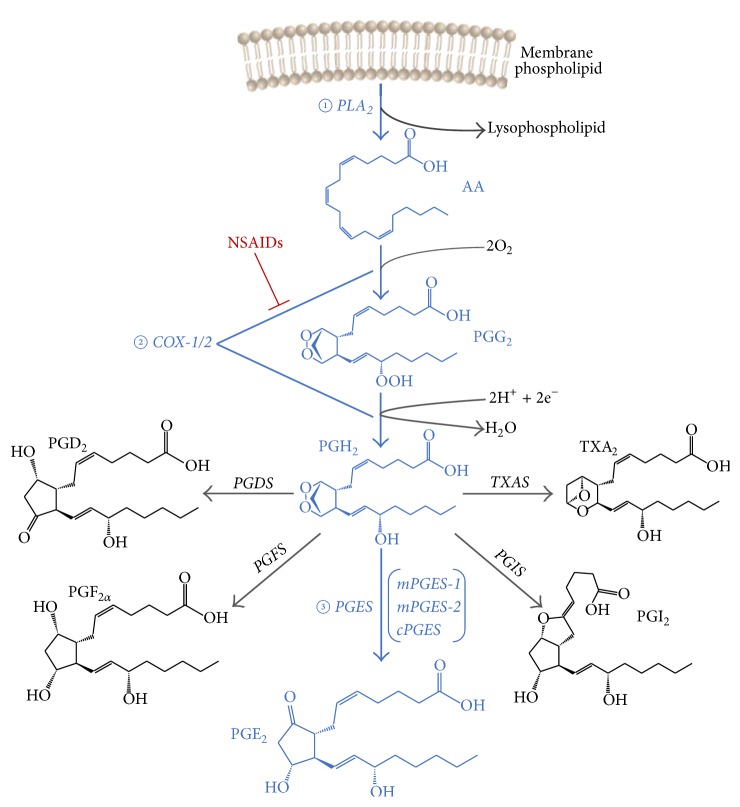
Biosynthesis pathway of prostaglandin E_2_.

PGE_2_ is synthesized via three sequential enzymatic reactions (Figure 1). Firstly, arachidonic acid (AA) is released from membrane phospholipids by phospholipase A_2_ (cPLA_2_); then, AA is converted into the unstable endoperoxide intermediates PGG_2_ and PGH_2_ by cyclooxygenase-1 (COX-1) or COX-2. Finally, PGH_2_ is converted to PGE_2_ through three terminal PGE_2_ synthases, two membrane associated PGE_2_ synthases (mPGES-1 and mPGES-2) and a cytosolic (cPGES) PGE_2_ synthase [1]. COX-1 is usually constitutively expressed in most tissues and responsible for the basal production of PGE_2_ that is involved in homeostasis of various physiological functions, such as gastrointestinal and kidney maintenance. In contrast, the expression of COX-2 is very low in many tissues at baseline but is highly induced by proinflammatory factors, hormones, and growth factors. Its role in the production of inflammatory PGE_2_ and probably prostacyclin (PGI_2_) provided the rationale for the development of COX-2 selective NSAIDs, such as celecoxib, rofecoxib, and valdecoxib, for the management of pyrexia, relief of pain, and alleviation of inflammation with less gastrointestinal side effects [2]. However, placebo-controlled trials revealed that these drugs predisposed patients to a series of cardiovascular hazards, including hypertension, stroke, myocardial infarction, heart failure, and sudden cardiac death, affecting ~1-2% of patients exposed per year [3]. For example, the VIGOR trial showed a 0.4% increase in myocardial infarction in the patients given COX-2 selective NSAID rofecoxib, but only 0.1% increase for those given the nonselective naproxen [4]. The molecular mechanism underlying these complications has been variously studied. The leading explanation is that COX-2 inhibition depresses PGI_2_ formation in the vasculature which restrains platelet activation by prothrombotic stimuli. Inhibition of this mediator increases the likelihood of thrombotic events, hypertension, and heart failure particularly in patients at elevated cardiovascular risk [5].

Among the three PGESs, mPGES-2 and cPGES are constitutively expressed and had been thought to be responsible for the baseline PGE_2_ production. The baseline expression of mPGES-1 is relatively low in most tissues, while in response to acute and chronic inflammatory stimuli, mPGES-1 is upregulated and functionally coupled with COX-2 to mediate inflammatory PGE_2_ production [6]. The human mPGES-1 gene is localized to chromosome 9q34.3 and contains 3 exons and spans 14.8 kb. The protein consists of 152 amino acid residues with about 80% similarity to the enzyme in mouse, rat, or cow [7]. Owing to the undesirable effects of COX-2 selective inhibitors, interest has been focused on mPGES-1 as an alternative target for the development of analgesics and anti-inflammatory drugs [8]. The thought was that the analgesic efficacy would be largely, if not totally, conserved by PGE_2_ suppression while most of the cardiovascular risk would be minimized by conserving or even boosting the cardioprotective PGI_2_ production [9]. Indeed, global or myeloid specific deletion of mPGES-1 has proven efficacy in restraining atherogenesis, attenuating the proliferative response to vascular injury, and limiting aortic aneurysm formation [10–14]. However, there are also some opposite findings that global or macrophage mPGES-1 inhibition might adversely interfere with cardiac remodeling [15] and elicit hypertension [16, 17], which complicates the development of mPGES-1 inhibitors.

PGE_2_ acts on four specific G-protein-coupled receptors (GPCR) subtypes, termed EP1–4. Activation of these receptors by PGE_2_ or artificial compounds stimulates distinct signal transduction pathways and mediates various biological functions [18]. The EP1 receptor couples to G_q_-proteins to increase intracellular Ca^2+^ concentration. The EP2 and EP4 receptors couple to G_s_-proteins and evoke an increase in intracellular cAMP concentration. EP3 receptor mainly couples to G_i_-proteins to decrease cAMP production, while it has at least 8 variants which may activate other different signaling pathways, for example, elevate intracellular Ca^2+^, or activate the small G-protein Rho. The involvement of the four PGE_2_ receptors in cardiovascular function has been studied with various genetic deletion approaches or small molecule agonists or antagonists, and the conclusions varied [19].

## 2. mPGES-1 and Blood Pressure 

A change in blood pressure is a pronounced risk signal that reflects the increased cardiovascular hazard from NSAIDs. As the most promising target for next generation of analgesic and anesthetic drugs, it is of critical significance to understand the role of mPGES-1 in blood pressure regulation. Several lines of evidence point to no differences in blood pressure between mPGES-1-deficient and wild-type mice. Cheng et al. and Francois et al. reported that deletion of mPGES-1 failed to elevate blood pressure in mice fed either a normal or a high-salt diet [13, 20]. Angiotensin II induced hypertension was also uninfluenced by mPGES-1 deletion in hyperlipidemic mice [11, 21]. These results are somewhat inconsistent with the findings from Jia et al. and Zhang et al. who did note that mPGES-1 deletion augmented the hypertensive response to both salt loading and angiotensin II infusion [22, 23]. Interestingly, using Cre-LoxP mediated cell specific gene depletion, our own data illustrated that neither myeloid nor vascular cell mPGES-1 play a major role in blood pressure homeostasis, at least at baseline and hyperlipidemia conditions [14]. Howbeit, based on bone marrow transplant technology, the most recent JCI paper demonstrated that COX-2-mPGES-1 derived PGE_2_ in hematopoietic cells, especially macrophages, did contribute to blood pressure buffering in response to chronically increased dietary salt [16, 24]. The mechanism for these discrepancies has not been fully explored but seems to be associated with the differences in genetic background and differences in the experimental protocols [25]. Nevertheless, whether mPGES-1 inhibition will have a safety blood pressure profile compared to COX-2 selective NSAIDs will have to be evaluated further in clinical studies.

## 3. EP Receptors and Blood Pressure 

PGE_2_ has been demonstrated to act as either vasodilator or vasoconstrictor depending on its binding to distinct EP receptors. The functional balance of pressor and depressor receptors activated by PGE_2_ plays an important role in the overall maintenance of normal blood pressure, while an imbalance may be a risk factor for development of essential hypertension [26].

Generally, activation of the EP1 and EP3 receptors is vasoconstrictive [27]. It has been clearly documented that genetic disruption of EP1 or EP3 receptors blunted the hypertensive response to acute or chronic AngII infusion; the pressor responses to EP1/EP3 agonist sulprostone were abolished in EP1 or EP3 null mice [28–31]. Similarly, pharmacologic blockade of EP1 reduces blood pressure in the spontaneously hypertensive rat [28] and restrains the development of hypertension in mice with type 2 diabetes [32]. Various EP1 or EP3 antagonists have proven efficacy in blunting the constricting effect of AngII on arterial rings ex vivo [28, 29, 32]. Furthermore, EP1 deficient mice showed reduced vasodepressor response to exogenous PGE_2_ administration; a more prolonged reduction in mean arterial pressure to PGE_2_ infusion was displayed in EP3 null mice, although no difference in the magnitude of the depressor response was observed [28, 29]. In addition, activation of EP3 receptor is verified to be responsible for the sympathetic responses to central PGE_2_ [33, 34], and central AngII-driven sympathetic responses are mediated by brain EP1 activation [30]. Most recently, Lu et al. demonstrated that EP3 activation can facilitate hypoxia-induced vascular remodeling and pulmonary hypertension in mice [35]. The role of EP1 activation in sympathoexcitatory responses and pulmonary hypertension is still not clear.

Contrary to EP1 and EP3, activation of the EP2 and EP4 receptors is thought to be vasodilatory. Deletion of EP2 in mice led to slightly elevated baseline systolic blood pressure, and the pressor response to PGE_2_ infusion was unmasked [26, 36, 37]. When challenged with a high-salt diet, the EP2 knockout mice developed profound but reversible hypertension [36]. However, it is somewhat contentious because the PGE_2_ caused relaxations were unchanged in rings from EP2 null mice, and only limited relaxation was observed when treated with EP2 specific agonist, butaprost [38]. Tilley et al. even reported systemic hypotension in EP2 knockout mice [39]. This distinction perhaps again reflects differences in the genetic background (C57BL/6 versus 129/SvEv). Evidence of vasodilatory function of EP4 is supported by using the aortic ring preparation, where both EP4 deletion and administration of EP4 antagonist abolished the vessel relaxation effect of PGE_2_ [38]. The maximal vasodepressor effect of PGE_2_ in vivo was also significantly buffered in EP4 deficient mice, but only in females [37]. It is of note that Zhang et al. showed that although EP4-selective agonist prostaglandin E1-OH functioned as a vasodilator, activation of EP4 receptor alone failed to battle the pressor effect of EP3 in EP2 deficient mice [26]. This suggested that the vasodepressive effects of EP2 and EP4 receptors are predominant, which leads to the net effect of blood pressure depression of PGE_2_. Surprisingly, a prohypertensive action of EP4 activation is reported most recently, where Wang et al. showed that inactivation of EP4 would significantly lower AngII induced hypertension in Sprague-Dawley rats [40]. Mechanically, this effect might be due to the inhibition of the protein expression of renal (pro)renin receptor and the consequent suppression of local renin-angiotensin system.

**Figure 2:**
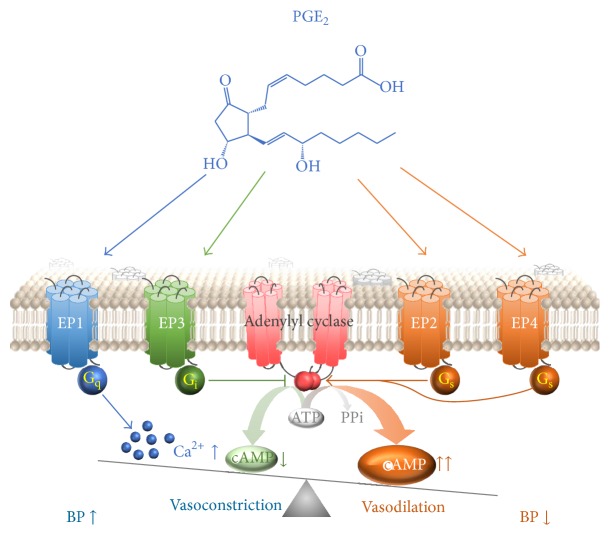
Function of EP receptors in blood pressure regulation. PGE_2_ increases vascular tone and thus blood pressure through EP1-mediated Ca^2+^ influx and EP3-mediated inhibition of cAMP synthesis, while it lowers blood pressure via EP2- and EP4-mediated activation of adenylyl cyclase and cAMP synthesis.

Taken together, despite an overall prohypertensive role of the EP1 and EP3 receptors and vasodepressor action of the EP2 and EP4 receptors (Figure 2), effects of PGE_2_ on blood pressure regulation are a complex interplay between each receptor subtype expression and other various factors in the genetic background. PGE_2_ in water and sodium reabsorption and renal hemodynamics plays vital roles in blood pressure control as well, while this is beyond the scope of the current review. Nevertheless, selective targeting PGE_2_ receptors may hold promise to develop into novel therapies for the management of hypertension and stroke.

## 4. mPGES-1 and Atherosclerosis and Other Inflammatory Vascular Diseases 

Deletion or inhibition of COX-2 has been shown variously to promote, postpone, or leave the development of atherosclerosis unaltered in diverse rodent models [41–43]. This perhaps reflects the contrasting biological effects of different COX-2 products formed by distinct cells during disease evolution. Indeed, the functional importance of COX-2 in individual vascular cell types on atherogenesis has been extensively studied. For instance, myeloid COX-2 promotes while vascular COX-2 restrains atherogenesis in diet induced hyperlipidemic mice [44, 45]. Similarly, despite the favorable atheroprotective and antianeurysm observation of global deletion of mPGES-1 in hyperlipidemic mice [11, 12], tissue-dependent consequences of mPGES-1 blockade were observed in cellular specific knockout mice. Recently, we found that myeloid cell mPGES-1 depletion restrains the initiation and early development of atherosclerosis, which is concomitant with a reduction in iNOS-mediated oxidative stress. By contrast, disruption of mPGES-1 in vascular smooth muscle cells, endothelial cells, or both does not detectably alter atherogenesis in mice [10]. This data points to the therapeutic rationale for targeting macrophage mPGES-1 in atherosclerosis. Interestingly, unlike the global mPGES-1 KO mice, our data showed no evident alteration in urinary production of PGI_2_ when mPGES-1 is lacking in macrophages only [10]. This is theoretically attractive, since PGI_2_, which mediates pain and dominates even over PGE_2_ in some mouse models of analgesia, might undermine the analgesic efficacy of mPGES-1 inhibition.

A similar case was observed in a wire-induced vascular injury model. Global deletion of mPGES-1 attenuated proliferation responses to wire injury via both suppression of PGE_2_ and product rediversion to PGI_2_ [46]. However, phenotypic divergence was observed when mPGES-1 was selectively deleted in given cell types [14]. Thus, the proliferative response to vascular injury was attenuated by myeloid cell mPGES-1 depletion, whereas it was promoted by EC and VSMC mPGES-1 deletion. In this case, the results were attributable to differential consequences of EP activation in these two cell types, rather than contrasting products of PGH_2_ diversion.

At all events, these observations raise the possibility that the broader cardiovascular efficacies observed with global mPGES-1 deletion might be conserved by targeting myeloid mPGES-1 and that targeting macrophage mPGES-1 may be a strategy to further refine efficacy while limiting adverse effects attributable to enzyme inhibition in other tissues. Despite these promising results, given that people who received NSAIDs are usually already suffering coronary stenosis or an atherosclerotic disease, it remains a challenge that such an inhibitor caused reversal of the established atherosclerosis rather than retarding its development.

## 5. EP Receptors and Atherosclerosis and Other Inflammatory Vascular Diseases 

Accumulating evidence demonstrated that inflammation plays a central role in the cascade of events that result in atherosclerotic plaque erosion and rupture, in which the roles of the four EP receptors have been extremely studied.

EP4 was considered as the most abundant PGE_2_ receptor expressed in vulnerable human atherosclerotic lesions [47]. Studies regarding the role of EP4 in atherosclerogenesis are variable. EP4 overexpression was associated with enhanced culprit matrix metalloproteinases expression and deteriorated inflammatory reaction in atherosclerotic plaques [47]. EP4 deficiency showed suppressed early atherosclerosis by promoting macrophage apoptosis [48]. Both pharmacological and genetic EP4 inhibition displayed efficiency in attenuating abdominal aortic aneurism formation in both mouse and human models [49–52]. However, in contrast to these observations, Tang et al. reported that deficiency of EP4 on bone marrow-derived cells had little effect on plaque size or morphology in early atherosclerosis but accelerated local inflammation and altered lesion composition at later stages of atherosclerosis [53]. In addition, augmented elastin fragmentation and exacerbated aneurism formation also presented in bone marrow EP4 deficient mice [54]. The discrepancy in these results could result from differences in experimental protocols or differences in genetic background among the strains used in these experiments; the pathophysiologic importance of PGE_2_-EP4 pathway in experimental atherosclerosis or aneurysm formation still merits further investigation.

Activation of EP2 exerts both proinflammatory and anti-inflammatory effects in atherosclerotic plaques. Li et al. demonstrated that activation of EP2 receptor is involved in the adhesion of monocytes to endothelial cells of the vessel wall, one of the earliest events during atherosclerogenesis [55]. Also upregulation of EP2 was observed in abdominal aortic aneurysm [52]. On the contrary, studies using genetically modified mice revealed that deficiency of the EP2 receptor in mice promotes VSMC proliferation and migration and augments neointimal hyperplasia after vascular injury, suggesting that activation of EP2 may have potential implications in treating pathological vascular remodeling, such as atherosclerosis and restenosis [56].

The role of EP1 and EP3 receptors in atherosclerosis received less attention. Although Cipollone et al. did not detect EP1 or EP3 expression in human atherosclerotic plaques [47], others demonstrated that expression of EP1 and EP3 is mainly located in macrophages of the plaque shoulder region [57]. A recent study showed that oxLDL suppresses EP3 expression in macrophages, thus impairing EP3-mediated anti-inflammatory and antiatherosclerotic effects [58]. Most recently, by screening various pharmacological inhibitors, Zhang et al. identified that inhibition of EP3, especially its *α* and *β* splice variants, impaired VSMC migration and restricted vascular neointimal hyperplasia, whereas overexpression of EP3*α* and EP3*β* aggravated neointima formation [59]. However, the direct effect of EP1 and EP3 on atherogenesis needs to be illustrated.

## 6. mPGES-1 and Thrombosis

Thrombosis is the most pronounced risk signal associated with NSAIDs [60]. Inhibition of COX-2 or deletion of IP (PGI_2_ receptor) significantly accelerated thrombogenesis, reflected by shortened time to vascular occlusion after photochemical injury of the carotid artery and reduced thrombogenesis after laser-induced cremaster arterioles injury [5]; however, these effects were not observed in either global or myeloid cell mPGES-1 knockout mice [10, 13]. Effects on both augmented PGI_2_ and suppressed PGE_2_ might be relevant to this beneficial phenotype: PGI_2_ restrains thrombogenesis, while PGE_2_ elicits platelet aggregation at low concentrations via EP3 [61]. Howbeit, considering the potential significance of genetic and environmental factors on blood pressure response to mPGES-1 deficiency [25], it is a prerequisite to confirm these favorable phenotypes in other genetic backgrounds and eventually in clinic studies.

## 7. EP Receptors and Thrombosis

PGE_2_ has been reported to exhibit a biphasic effect on platelet aggregation depending on its concentration. It potentiates and inhibits platelet aggregation at low and high concentrations, respectively. While EP1 expression is lacking, the other three PGE_2_ receptors, EP2, EP3, and EP4, are all expressed in platelets and the expression level of EP3 is much higher than EP2 and EP4. The contribution of these three receptors to PGE_2_ induced platelet aggregation and thrombosis is well studied. In detail, EP3 mediates the proaggregatory effect of PGE_2_. EP3 agonists had shown concentration-dependent potentiation of platelet aggregation in vitro [61]. In vivo, the EP3 gene depletion mice showed significantly prolonged tail bleeding time and when challenged with arachidonic acid, the lung thrombus formation and mortality both attenuated in the EP3^−/−^ mice [62]. In addition, by mechanical rupture of the plaque with scratching in a murine model, Gross et al. showed that the atherothrombosis was drastically decreased when there was a lack of EP3 in platelets [63]. Indeed, DG-041, a direct-acting EP3 antagonist, has been considered as an effective antiplatelet and antiatherothrombosis drug without increasing bleeding risk [64, 65]. In contrast, the EP2 and EP4 signaling mediates the anti-aggregatory effects of PGE_2_, albeit the prostacyclin receptor (IP) plays the predominant inhibitory role at higher PGE_2_ concentrations. Notably, these inhibitory effects of EP2 and EP4 might only be efficient when EP3 receptor is absent; thus, unaltered inhibitory effects of PGE_2_ in EP2 or EP4 single knockout platelets were observed, while in EP3 and IP double deficient platelets the inhibitory effect was augmented [66]. Nevertheless, PGE_2_ sensitizes platelets to their agonists such as thrombin or collagen through the activation of its EP3 receptor, while PGE_2_ inhibits platelet activity through EP2 and EP4 receptors [66]. The net result of these opposing actions is that the stimulating effect of EP3 overcomes the inhibiting effects of EP2 and/or EP4 and leads to platelet aggregation and potentiates thrombosis [67]. Selective blockade of the EP3 activity and/or activation of EP2 or EP4 are rational strategies for developing novel antiplatelet agents and preventing thrombogenesis.

## 8. mPGES-1 and Myocardial Remodeling

The role of mPGES-1 inhibition in cardiac remodeling is complex. Unlike COX-2 inhibition, loss of mPGES-1 avoided the post-MI death after coronary occlusion in mice [68]. Notably, the loss of mPGES-1 did not significantly affect the circulatory PGE_2_ level, while it was accompanied with an induction of PGI_2_ after myocardial infarction (MI). Therefore, it is possible that a redirected synthesis to PGI_2_ compensates the loss of mPGES-1 and offers the benefit upon acute ischemia. This is also supported by the finding that pretreatment of IP antagonist in mice lacking mPGES-1 increased myocardial damage and reduced postischemia survival [69]. However, although global mPGES-1 deletion does not increase mortality, it does adversely influence myocardial remodeling after coronary artery ligation in mice. Degousee et al. showed that, twenty-eight days after MI, mPGES-1 KO mice developed more severe pathological left ventricle (LV) remodeling compared to WT, including eccentric cardiomyocyte hypertrophy, impaired LV systolic and diastolic function, LV dilation, and elevated LV end-diastolic pressure [15]. In this case, it seems that mPGES-1-derived PGE_2_ has beneficial effects on myocardial remodeling. It was found that the induction of the mPGES-1 protein was mainly produced from inflammatory cells of the heart after myocardial infarction [15]. Thus, by using the bone marrow transplant approach, Degousee's group further demonstrated that inactivation of mPGES-1 in bone marrow-derived leukocytes fully replicated the deleterious remodeling phenotypes and decreased the post-MI survival [70]. Surprisingly, although mPGES-1 was lacking in bone marrow leukocytes, an even higher level of PGE_2_ was detected in infarct and viable myocardium. This probably comes from the induction of mPGES-1 activity in cardiac fibroblasts in the chimera mice. However, this evokes an interesting paradox: either boosting or decreasing PGE_2_ is associated with worse LV remodeling and function. One possibility is that the deletion of mPGES-1 globally or specifically in the bone marrows renders the PGH_2_ substrate available for diversion to various other PG synthases. Secondly, the distinct expression or activation of the four PGE_2_ receptors in myocardium might result in varied biological effects of PGE_2_ in these two models. Nevertheless, more work is required to refine the role of PGE_2_ in bone marrow-derived leukocytes and in fibroblasts in mediating the remote control of myocardial remodeling. Indeed, we have new evidence that lacking mPGES-1 in myeloid cells, particularly in macrophages, promotes post-MI survival, while no detectable adverse influence on post-MI remodeling was observed (unpublished data).

## 9. EP Receptors and Myocardial Remodeling

Although the expression of the four EP receptors in heart varies among studies, an abundant expression of the EP4 mRNA has been reported in the heart with acute myocardial infarction [71]. Targeting EP4 has garnered much interest in treating myocardial infarction and heart failure. Indeed, administration of an EP4 agonist effectively reduced acute cardiac rejection, protected against allograft rejection, and prolonged allograft survival in mice by suppressing of inflammation during cardiac transplantation [72]. EP4 agonists also showed evidence to protect reperfused myocardium from ischemic injury, inactivation of EP4 aggravated myocardial remodeling, and impaired cardiac function in vivo [71, 73]. On the contrary, activation of EP4 mediated PGE_2_-induced cardiac myocyte hypertrophy in vitro [74]. Qian et al. showed that the cardiomyocytes-specific EP4 knockout mice had decreased cardiac hypertrophy and improved cardiac fibrosis while accompanied with impaired heart function outcome [75]. In this case, the myocyte EP4 induced hypertrophy seems to be cardioprotective, while the opposite effects of EP4 expression in other cell types, for example, fibroblasts and macrophages, might be another explanation. Thereby, the cell specific role of EP4 receptor in cardiac remodeling is further required. With regard to EP3, although several reports suggested a cardioprotective effect of EP3 agonists against ischemia/reperfusion injury in rodents and pigs [76–78], others demonstrated that overexpression of EP3 might activate calcineurin and promote hypertrophy after ischemia reperfusion [79]. These results suggest a dual involvement of the EP3 subtype in both cardioprotection and hypertrophy, which might be attributable to the expression diversity of EP3 isoforms in heart tissue. Most recently, Liu et al. suggested the necessity of PGE_2_-EP3 signaling in maintaining the normal growth and development of the heart, and they found that lacking EP3 receptor in mice would foster eccentric cardiac hypertrophy and fibrosis in 16–18-week-old mice even at resting condition [80]. Therefore, EP3 presents a potential therapeutic target for cardiac eccentric hypertrophy and cardiac remodeling. Unlike EP3 and EP4, the role of EP1 and EP2 receptors in cardiac remodeling is less studied. In vitro data showed that PGE_2_ stimulates cardiac fibroblast proliferation via EP1 [81] and mediates the effect of postischemic coronary effluent on Ca^2+^ transients and systolic cell shortening by EP4 and EP2 activation [82]. However, there is still no direct in vivo evidence showing that EP1 and EP2 are significantly involved in cardiac remodeling and this warrants further investigation.

## 10. Summary

In summary, growing evidence has illustrated the promising prospect of targeting mPGES-1, especially in macrophages, in inflammatory cardiovascular diseases, albeit further research is in need to fully pinpoint the effects and the side effects panorama of inhibiting mPGES-1. On the other hand, comprehensive data displayed a quite diverse, disease-specific contribution of individual EP receptors to cardiovascular health and diseases. More work is required to clarify the controversies and gain insight into the precise contribution of targeting each receptor. So far, specific inhibitors of mPGES-1 have advanced into clinical trials and various EP agonists and antagonists are pursued as alternative approaches to COX-2 inhibition. However, millions of patients worldwide are regular consumers of NSAIDs for pain relief and many of them are seniors (up to 40% of people 65 and older take NSAID daily)—a group already likely to have cardiovascular diseases. Thus, even the small incremental risk (~1-2%) of cardiovascular events caused by NSAIDs has been of concern. The future aim is to develop a class of drugs that not only afford pain relief but also have cardiovascular efficacy.
